# Straight or fractured? The DIDCO lifeline framework: a structured multidimensional model for psychiatric work capacity evaluation

**DOI:** 10.3389/fpsyt.2026.1877131

**Published:** 2026-07-15

**Authors:** Cristian Damsa

**Affiliations:** Centre Médical Lancy-Bachet, Grand-Lancy, Switzerland

**Keywords:** activities of daily living, disability evaluation, forensic psychiatry, international classification of functioning, disability and health, mental disorders, rehabilitation

## Abstract

Psychiatric work capacity evaluation remains characterized by substantial inter-rater variability and limited transparency in medico-legal contexts. While several validated dimensions relevant to work capacity assessment already exist—including diagnostic severity, functional impairment, ecological functioning, and occupational demands—psychiatry still lacks an integrated and visually structured framework allowing coherent synthesis of these elements in routine expert practice. This article proposes the DIDCO Lifeline framework, a multidimensional graphical model designed to support psychiatric work capacity evaluation through structured integration of five domains: diagnostic severity, ICF-based functional impairment, daily-life functioning, clinical coherence and plausibility, and occupational demands. Based on long-term clinical and medico-legal practice, the framework combines a structured classification table with a continuous graphical “lifeline” representation. Within this model, coherent clinical presentations generate relatively continuous profiles, whereas marked discontinuities or “fractures” between domains may indicate inconsistencies requiring further clinical interpretation and reassessment. The framework is not intended to introduce a new psychometric construct, but rather to provide practical organizational and communication tool integrating already established clinical dimensions into a transparent and structured representation. The DIDCO Lifeline may facilitate communication between treating psychiatrists, experts, insurers, and legal authorities by making the underlying clinical reasoning more explicit and visually accessible. The model also provides practical support for structured expert assessment in complex psychiatric disability evaluations.

## 1 Introduction

Psychiatric disorders are among the leading causes of work incapacity worldwide, yet their evaluation remains one of the least standardized domains in clinical and occupational practice. Determining work capacity in psychiatry requires integrating multiple sources of information, including symptom severity, functional limitations, contextual factors, and behavioral observations. However, in the absence of a structured framework, this integration often remains implicit, leading to substantial variability across clinicians.

Empirical studies have demonstrated limited inter-rater reliability in psychiatric work capacity assessments. The RELY (Reliable Disability Evaluation in Psychiatry) studies reported only moderate agreement between experts, with intraclass correlation coefficients around 0.4 for work capacity estimates (1). Such variability is problematic and highlights the need for more transparent and structured multidimensional clinical reasoning in practice.

Current approaches to psychiatric work capacity evaluation rely on several partially overlapping frameworks. Diagnostic classifications (DSM and ICD) provide standardized descriptions of mental disorders but do not directly translate into functional consequences. Functional assessment tools, such as the Mini-ICF-APP (2), offer structured evaluation of activity and participation limitations and have demonstrated reliability and clinical relevance in psychiatric work disability evaluations (3). However, these dimensions are often considered separately and are not systematically integrated into a unified and visually structured representation of clinical reasoning.

In contrast, neuropsychological practice routinely combines cognitive findings, behavioral observations, functional consequences, and symptom validity assessment into structured profiles that facilitate interpretation and communication of expert conclusions (4). Such approaches help clinicians organize complex information into coherent and clinically interpretable patterns.

Psychiatric work capacity evaluations similarly require continuous integration of diagnostic severity, functional impairment, real-life functioning, coherence and plausibility, and occupational demands. Yet psychiatry still lacks a practical framework allowing these dimensions to be visually synthesized into an explicit and shared representation.

The present work proposes the DIDCO Lifeline framework, a multidimensional graphical model designed to support psychiatric work capacity evaluation through structured integration of five clinically relevant domains. Rather than introducing a new psychometric construct, the framework aims to provide a practical organizational and communication tool that facilitates transparency, coherence assessment, and interdisciplinary dialogue between treating psychiatrists, experts, insurers, and legal authorities. By combining a structured classification table with a continuous graphical “lifeline” representation, the model seeks to make clinical reasoning more explicit, coherent, and visually accessible in routine psychiatric expert assessment.

The DIDCO Lifeline framework is conceptually situated within biopsychosocial and ICF-oriented models of disability, while also drawing from multidimensional approaches used in neuropsychological assessment and contemporary contextual models of work disability.

## 2 Methods

### 2.1 Conceptual development

The proposed framework was developed through long-term clinical and medico-legal practice in psychiatry over approximately 25 years. During this period, the author continuously integrated insights from the scientific literature with real-world clinical observations related to work capacity evaluation, while seeking to improve inter-rater consistency in psychiatric expert assessment, an issue previously explored in published work on inter-judge concordance and guideline use in disability expertise (5). This work proposes a conceptual and practice-based framework derived from long-term clinical and medico-legal experience rather than a formal empirical or psychometric study.

In addition, the model was progressively refined through structured intervision over approximately 15 years, involving systematic discussion of a large number of psychiatric expert assessments (estimated at around 10,000 cases). These discussions focused on the interpretation of clinical findings, functional limitations, coherence of presentations, and work capacity estimations, with particular attention to recurrent discrepancies in clinical reasoning between experts.

The objective of this iterative process was not to develop a new diagnostic instrument, but rather to organize clinically relevant dimensions already used in routine psychiatric assessment into a more explicit, coherent, and visually structured framework.

Over time, repeated discrepancies between expert assessments were systematically analyzed, particularly regarding the interpretation of symptom severity, functional impairment, ecological functioning, and occupational impact. This longitudinal process progressively led to the emergence of a multidimensional organizational model integrating five core domains into a continuous graphical representation.

The current framework represents the result of this progressive clinical refinement and is intended as a practical support tool for structuring clinical reasoning and facilitating communication across medico-legal contexts. The framework does not aim to replace clinical judgment but to provide a more transparent and visually accessible integration of clinically relevant information.

### 2.2 Conceptual approach

The framework was developed using an inductive approach combining clinical experience, case-based reasoning, and continuous comparison with existing models of functional assessment and work capacity evaluation.

Particular attention was given to situations characterized by discrepancies between reported symptoms, observed behavior, daily functioning, and occupational consequences, as such situations were repeatedly identified as a major source of uncertainty and disagreement in psychiatric expert assessments.

### 2.3 Selection of dimensions

Based on this process, five clinically relevant domains were identified for structured integration within the DIDCO Lifeline framework: diagnostic severity, functional impairment, real-life functioning, clinical coherence and plausibility, and occupational context.

The operational organization and graphical integration of these dimensions are presented in the Results section.

### 2.4 Analytical principles

The framework was developed on the basis of three core principles. First, psychiatric work capacity evaluation requires integration of multiple sources of clinical information in order to capture the complexity of psychiatric presentations. Second, particular importance was attributed to the coherence and plausibility of clinical findings across domains. Third, work capacity was conceptualized as context-dependent and therefore inseparable from occupational demands and environmental constraints.

The resulting graphical “lifeline” representation was designed to facilitate visual synthesis of these dimensions and to support explicit communication of clinical reasoning in routine psychiatric expert assessment.

## 3 Results

### 3.1 Structure of the DIDCO lifeline framework

The DIDCO Lifeline framework integrates five clinically relevant dimensions into a unified graphical representation designed to support psychiatric work capacity evaluation. The framework combines diagnostic severity, functional impairment, real-life functioning, clinical coherence and plausibility, and occupational demands into a continuous multidimensional profile intended to facilitate explicit clinical reasoning and communication across medico-legal contexts.

Each dimension is rated on a four-level scale (0–3), reflecting increasing levels of severity or impact. The coherence dimension constitutes a specific case, as it reflects the degree of consistency and plausibility across domains rather than severity itself.

### 3.2 Multidimensional classification table

The framework includes a structured multidimensional table integrating the five domains into a single visual and organizational support tool (**Table 1**). The purpose of this table is not to generate automatic conclusions, but rather to facilitate coherent integration of clinically relevant information during psychiatric work capacity evaluation.

**Table 1:** Multidimensional classification of psychiatric work capacity.

Level	Diagnostic severity	Typical daily functioning	Functional impairment (Mini-ICF)	Clinical coherence	Job demands	Work capacity (current job)	Work capacity (adapted job)	Prognosis
0	No disorder or invalid presentation	Normal functioning	No limitation	Major incoherence / invalid presentation	All levels	100%	100%	No psychiatric limitation
1	Mild disorder / adjustment disorder	Slightly reduced but structured functioning	1–3 mild limitations	Partial to good coherence	Low to moderate	70–100% (temporary incapacity possible)	80–100%	Favorable
2	Moderate disorder	Reduced activity, avoidance, fatigability	Multiple domains affected	Good coherence	Moderate to high	40–70% (context-dependent incapacity possible)	50–80%	Intermediate
3	Severe disorder	Loss of autonomy, major functional restriction	Global impairment	High coherence required for validity	All levels	0–40%	0–40%	Unfavorable

The table presents a structured multidimensional framework integrating five core domains (DIDCO): diagnostic severity (D), ICF-based functional impairment (I; activity and participation), daily-life functioning (D; typical day), clinical consistency and plausibility (C), and occupational demands (O). Clinical severity is estimated through convergence across domains rather than diagnosis alone. Clinical coherence reflects the degree of consistency between reported symptoms, observed behaviour, and functional outcomes and serves as a central interpretative dimension within the framework. Work capacity is considered in both the current and an adapted occupational context, reflecting the interaction between individual impairment and job demands. The proposed work-capacity ranges are intended as indicative reference points supporting structured clinical reasoning and should not replace individualized clinical assessment. Marked incoherence may require cautious interpretation of the clinical presentation from a medico-legal perspective, particularly when the available findings do not form a sufficiently coherent and clinically interpretable whole. D, Diagnostic severity; I, ICF-based functional impairment (activity and participation); D, Daily-life functioning (typical day); C, Clinical consistency and plausibility; O, Occupational demands.

The table allows clinicians to visualize convergence or divergence across domains and to organize clinical reasoning in a more explicit and transparent manner within both current and adapted occupational contexts.

### 3.3 Clinical coherence and plausibility

Clinical coherence constitutes the central interpretative principle of the DIDCO Lifeline framework. The assessment focuses on the degree of consistency between reported symptoms, observed behavior, ecological functioning, and occupational consequences.

Incoherent or markedly discontinuous profiles may emerge in situations where severe subjective complaints coexist with preserved autonomy or where reported limitations are not consistent with observed functioning. Such discrepancies may reflect amplification, exaggeration, simulation, or other factors requiring careful clinical interpretation (6).

Rather than functioning as a formal validity test, the coherence dimension is intended to support structured clinical interpretation by making discrepancies visually explicit within the overall profile.

### 3.4 Graphical lifeline representation

**Figure 1:**
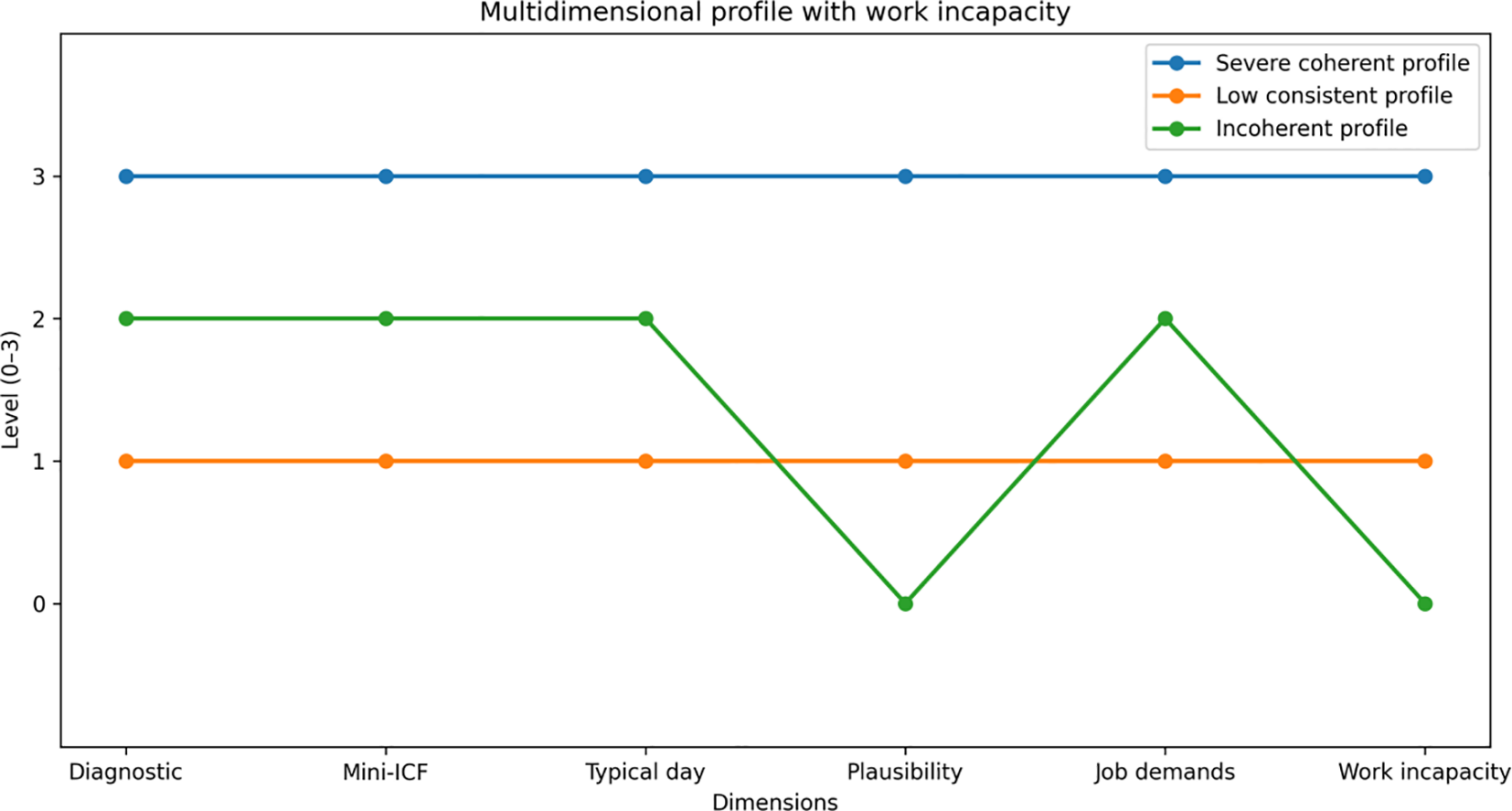
Multidimensional DIDCO Lifeline profile for psychiatric work capacity evaluation.

The central feature of the framework is the graphical “lifeline” representation of the clinical profile (**Figure 1**). Each dimension is plotted sequentially on a vertical scale (0–3), and the resulting points are connected to generate a continuous multidimensional line.

Relatively continuous and convergent profiles tend to reflect coherent clinical presentations, whereas marked discontinuities or “fractures” between dimensions may indicate clinically relevant inconsistencies requiring further interpretation or reassessment.

The graphical profile is intended to facilitate rapid visual synthesis of complex clinical information and to support communication between treating psychiatrists, experts, insurers, and legal authorities. By making the structure of clinical reasoning more explicit and visually accessible, the DIDCO Lifeline aims to improve transparency in psychiatric work capacity evaluation. An example of a severe convergent profile (red line) illustrates uniformly elevated levels across domains, consistent with a severe and globally impairing clinical presentation. An example of a severe convergent profile (blue line) illustrates mild and relatively homogeneous levels across domains, compatible with limited functional impact and preserved work capacity. An example of a discrepant profile (green line) illustrates moderate levels of diagnostic severity and functional impairment associated with increased real-life impact and occupational demands, but limited clinical consistency and plausibility. Such discrepancies may require cautious interpretation of the relationship between reported symptoms, observed functioning, and occupational consequences within the medico-legal evaluation process. The systematic use of multidimensional graphical profiles may facilitate explicit integration of clinically and legally relevant dimensions and support more transparent communication across expert assessments.

## 4 Discussion

The present work proposes the DIDCO Lifeline framework, a structured multidimensional representation designed to support psychiatric work capacity evaluation in clinical and medico-legal settings. Rather than introducing a new psychometric construct, the framework aims to organize clinically relevant dimensions already used in psychiatric assessment into a coherent and visually explicit representation.

A central contribution of the model lies in the integration of a graphical “lifeline” profile intended to facilitate visualization of convergence and discrepancies across domains (**Figure 1**). By making clinical reasoning more explicit and visually accessible, the framework may support communication between treating psychiatrists, experts, insurers, and legal authorities.

This approach is consistent with the principles emphasized in Swiss Federal Supreme Court jurisprudence, particularly ATF 141 V 281 (6), which highlighted the importance of structured evaluation of functional limitations, coherence, and real-life impact in psychiatric disability assessment. In this context, the DIDCO Lifeline framework may be understood as a practical graphical representation facilitating multidimensional clinical interpretation across the severity spectrum. The proposed graphical model provides a direct operationalization of these criteria. More broadly, the framework may be understood as a first step toward a standardized neuropsychology-like system (ASNP-equivalent) for psychiatric expertise, where structured profiles support both clinical reasoning and medico-legal decision-making (7).

This graded approach further complements the structured indicators introduced in subsequent Swiss case law (2017), which emphasized the structured evaluation of intermediate clinical presentations, particularly moderate depressive disorders. In practice, this jurisprudence progressively introduced a three-level logic involving clearly severe conditions, absence of sufficient severity, and intermediate situations requiring detailed multidimensional assessment, later extended to other conditions such as substance use disorders (2019) (6).

However, the practical integration of milder or heterogeneous presentations remains less explicitly structured in routine psychiatric expert assessment. In this context, the DIDCO Lifeline framework proposes a continuous and transdiagnostic graphical representation intended to facilitate integration of clinical severity, functional impairment, coherence, and occupational impact across the full spectrum of psychiatric presentations.

### 4.1 Addressing a structural gap in psychiatric evaluation

Psychiatric work capacity assessment remains inherently complex and variable. Unlike somatic conditions, psychiatric disorders involve subjective symptomatology, fluctuating clinical courses, and strong contextual influences. As a result, the translation of symptoms into functional consequences is often implicit and highly dependent on individual clinical judgment.

Empirical studies have consistently highlighted this variability, demonstrating only moderate agreement between experts when estimating work capacity. This limited agreement reflects not only clinical complexity but also the absence of a structured framework guiding the integration of diagnostic, functional, and contextual information. The lack of such a framework likely contributes directly to the persistent variability observed in psychiatric work capacity evaluations (8, 9).

The DIDCO Lifeline framework aims to address this structural gap by providing a structured and visually explicit representation integrating diagnostic, functional, behavioral, and contextual dimensions within a shared multidimensional profile.

### 4.2 Beyond diagnosis: the need for multidimensional integration

A central limitation of traditional approaches lies in the reliance on diagnostic classification as the primary determinant of work capacity. While diagnosis provides an essential clinical framework, it does not adequately capture functional consequences or real-world impact (8, 9).

The present model explicitly integrates functional impairment and real-life functioning, ensuring that evaluation is grounded in observable and ecologically valid data. This is consistent with prior work emphasizing the importance of activity and participation limitations in psychiatric disability assessment (10–12).

However, the key contribution of this framework lies not in the identification of new dimensions, but in their structured integration within a single operational system. By organizing these elements into a unified model, the framework facilitates more explicit and transparent multidimensional clinical reasoning through structured integration of heterogeneous clinical observations.

### 4.3 The role of clinical coherence as a validity mechanism

One of the most distinctive features of the proposed framework is the structured integration of clinical coherence across domains. In psychiatric evaluation, discrepancies between reported symptoms, observed behavior, daily functioning, and occupational consequences are common and frequently contribute to diagnostic uncertainty and inter-rater variability.

By incorporating coherence as an explicit dimension within the graphical profile, the DIDCO Lifeline framework aims to facilitate structured clinical interpretation of convergences and discrepancies across domains. Rather than functioning as a formal symptom validity test or diagnostic tool for simulation, the framework is intended to support transparent multidimensional clinical reasoning.

This pragmatic approach is conceptually consistent with principles used in neuropsychological assessment, where interpretation of findings relies on integration of behavioral, functional, and validity-related information within a coherent clinical framework.

### 4.4 Work capacity as a contextual and non-linear construct

Another important contribution of the framework is the explicit recognition that work capacity is not an intrinsic property of the individual. Instead, it emerges from the interaction between psychiatric impairment and occupational demands.

This perspective explains why similar clinical presentations may lead to different work capacity outcomes depending on the job context. It also justifies the systematic distinction between capacity in the current job and in an adapted occupational environment.

Furthermore, the model emphasizes the non-linear relationship between severity and work capacity, particularly in high-demand or safety-critical occupations. This non-linearity reflects real-world clinical practice and highlights the limitations of rigid or purely quantitative approaches.

One important limitation of the proposed framework concerns the restricted granularity of the four-level scaling system, particularly in intermediate clinical presentations where medico-legal decisions are often the most difficult. In such situations, work-capacity conclusions cannot be derived from isolated categorical levels but require multidimensional interpretation integrating functional impairment, ecological functioning, occupational demands, and coherence across domains. The proposed 0–3 ratings should therefore be understood as broad organizational anchors rather than precise quantitative thresholds.

### 4.5 Practical implications: toward a minimal common framework

From a practical perspective, the DIDCO Lifeline framework aims to function as a shared organizational and graphical language for psychiatric work capacity evaluation. By combining a structured multidimensional table with a continuous graphical profile, the framework facilitates explicit integration of diagnostic, functional, behavioral, and occupational dimensions within a single visual representation.

The graphical component plays a central role in this process. Relatively continuous profiles tend to reflect coherent clinical presentations, whereas marked discontinuities or “fractures” between dimensions may indicate clinically relevant inconsistencies requiring further interpretation.

Rather than replacing clinical judgment, the graphical profile is intended to support structured reflection, transparency, and interdisciplinary communication. By transforming implicit reasoning into a visually accessible representation, the DIDCO Lifeline may facilitate dialogue between treating psychiatrists, experts, insurers, and legal authorities in complex medico-legal situations.

### 4.6 Limitations and future directions

Several limitations should be acknowledged. The model is based on long-term clinical experience and conceptual integration rather than prospective empirical validation. Consequently, its impact on inter-rater reliability remains to be formally demonstrated.

In addition, while the framework provides structured guidance, it does not eliminate the need for clinical judgment. A degree of subjectivity remains inherent to psychiatric evaluation.

Future research should focus on prospective validation of the model, including formal assessment of inter-rater agreement and comparison with existing evaluation methods. Such validation studies could involve independent parallel evaluations of the same claimants by psychiatrists with different levels of expertise (for example junior and senior psychiatrists) in order to assess inter-rater agreement regarding multidimensional profiles, coherence interpretation, and work-capacity conclusions generated using the DIDCO Lifeline framework. Within our supervision and intervision network involving fourteen psychiatrists working in medico-legal assessment, including Swiss Insurance Medicine certified experts, preliminary interest has already been expressed regarding future pilot studies involving routine expert practice.

Such studies will be essential to determine the extent to which the proposed framework supports transparent and structured multidimensional clinical reasoning in practice.

### 4.7 Integration with existing functional and work capacity assessment frameworks

The proposed multidimensional framework can be situated within the broader field of functional and work capacity assessment, which includes established instruments such as WHODAS 2.0, the Global Assessment of Functioning, and conceptual models of work disability, as well as structured approaches such as Functional Capacity Evaluation.

WHODAS 2.0 represents a widely used and psychometrically validated instrument for the assessment of disability across domains of functioning. It provides a standardized description of activity limitations and participation restrictions and has demonstrated cross-cultural applicability and reliability. However, its primary function remains descriptive, and it does not directly provide a structured framework for translating impairment into work capacity conclusions in medico-legal contexts (10, 11).

The Global Assessment of Functioning (GAF) scale historically attempted to integrate symptom severity and functional impairment into a single score. Despite its clinical utility, concerns regarding its reliability and conceptual clarity have led to its removal from DSM-5, highlighting the limitations of unidimensional approaches to complex psychiatric presentations (12).

Contemporary models of work disability emphasize the interaction between health conditions, functional limitations, and contextual factors, particularly workplace demands and environmental constraints. These models have provided an important conceptual shift toward viewing work capacity as a dynamic and context-dependent construct (13). However, their application in clinical practice often remains heterogeneous and lacks a unified operational structure.

Functional Capacity Evaluation approaches, widely used in somatic medicine, offer structured and standardized assessment of functional abilities in relation to job demands. While these approaches provide objective data, their direct applicability to psychiatric conditions is limited by the inherent variability, subjectivity, and contextual sensitivity of psychiatric symptoms (14).

The present framework builds upon these existing approaches but differs in its objective. Rather than introducing new constructs, it seeks to integrate previously validated dimensions into a single structured and clinically applicable system. By combining diagnostic severity, functional impairment, real-life functioning, clinical coherence, and occupational demands within a unified classification, the model translates conceptual principles into an operational framework aimed at supporting more explicit and transparent multidimensional integration in expert assessments.

### 4.8 Internal consistency and clinical plausibility

A central element of the framework is the systematic evaluation of internal consistency across domains. In particular, the comparison between real-life functioning (e.g., description of a typical day) and structured functional assessment (such as Mini-ICF-APP) provides clinically relevant information regarding the plausibility of the overall presentation.

Discrepancies between these domains are not uncommon in psychiatric evaluations and require careful interpretation. Such inconsistencies may reflect fluctuations in symptom expression, contextual influences, or limitations in self-report accuracy. In medico-legal contexts, they may also raise questions regarding the reliability of the reported impairment (3, 15).

The present model does not aim to directly diagnose malingering but rather to promote a structured assessment of clinical coherence and plausibility, consistent with current approaches to symptom validity evaluation in psychiatric and neuropsychological practice.

### 4.9 Observational impact in clinical practice

Although the proposed framework has not yet been formally validated in prospective studies, its long-term use in routine medico-legal practice has been associated with a marked reduction in contestations of expert reports.

Over a period of approximately ten years, the systematic application of this structured approach was associated with a frequency of contested reports that has more than halved compared to prior unstructured evaluations. However, these observations remain anecdotal and cannot be interpreted as evidence of improved reliability or effectiveness. Formal prospective evaluation using appropriate methodological approaches will be necessary.

This evolution likely reflects the formalization of established clinical reasoning processes into a structured and communicable format, which better satisfies the evidentiary requirements of legal and insurance stakeholders.

This paper may reflect not the validation of a novel theoretical model, but rather the formalization of established clinical reasoning processes into a structured and communicable format.

### 4.10 Graphical representation as a shared framework

The graphical representation of the multidimensional profile constitutes an additional practical component of the model. By visually integrating all dimensions into a continuous profile, it facilitates the identification of convergence or discrepancies across domains.

Such representations may serve as a shared framework for communication between clinicians, experts, insurers, and legal authorities. In this sense, they may contribute to the development of a common language in psychiatric work capacity evaluation, improving both transparency and interpretability.

From a medico-legal perspective, structured and explicit multidimensional representations may strengthen the transparency and interpretability of psychiatric expert assessments by ensuring that diagnostic, functional, behavioral, and contextual dimensions are explicitly integrated within a coherent clinical framework, as illustrated in **Figure 1**.

This perspective is consistent with recent work in forensic psychiatry emphasizing explicit clinical reasoning competencies, structured psychomedicolegal analysis, and reproducible approaches to psychiatric disability evaluation rather than purely implicit expert judgment (16–20). In this context, the DIDCO Lifeline framework is not intended to function as a new psychometric instrument requiring immediate validation, but rather as a structured graphical support for multidimensional clinical reasoning, analogous to a cognitive “check panel” allowing rapid visualization of whether diagnostically, functionally, behaviorally, and contextually relevant dimensions have been coherently integrated within the expert assessment.

## 5 Conclusion

The DIDCO Lifeline framework proposes a structured multidimensional representation for psychiatric work capacity evaluation integrating diagnostic severity, functional impairment, real-life functioning, clinical coherence, and occupational demands within a continuous graphical profile.

Rather than functioning as a formal psychometric instrument, the framework is intended to support explicit clinical reasoning, visual synthesis of complex information, and interdisciplinary communication in medico-legal contexts.

By facilitating identification of coherent or “fractured” profiles across domains, the graphical lifeline representation may contribute to improved transparency and interpretability in psychiatric expert assessment. Future prospective studies will be necessary to evaluate its potential impact on consistency and clinical practice.

## Data Availability

The original contributions presented in the study are included in the article/supplementary material. Further inquiries can be directed to the corresponding author.
